# miR-181a-5p Inhibits Pyroptosis in Sepsis-Induced Acute Kidney Injury through Downregulation of NEK7

**DOI:** 10.1155/2022/1825490

**Published:** 2022-08-10

**Authors:** Meng Zhang, Deyuan Zhi, Jin Lin, Pei Liu, Yajun Wang, Meili Duan

**Affiliations:** Department of Critical Care Medicine, Beijing Friendship Hospital, Capital Medical University, No. 95 Yong'an Road, Xicheng District, Beijing 100050, China

## Abstract

Sepsis is a life-threatening organ dysfunction caused by the uncontrolled inflammation, easily affecting the kidney. Sepsis-induced acute kidney injury (S-AKI) has high morbidity and mortality, of which the pathophysiological mechanisms have not been completely illuminated, leading to nonspecific therapies. Specific microRNAs were related with the pathogenesis of AKI. However, only limited studies focused on the pyroptosis in the context of S-AKI. The in vitro LPS-induced HK-2 cell model and in vivo CLP-induced mouse model were established. qRT-PCR, Western blot, ELISA, and RNA pulldown were used for expression examination. Multiple biological databases were used for miRNA screening. H&E staining and IHC staining were performed. The LPS-induced HK-2 cells showed significantly increased (*P* < 0.01) fluorescence intensity of N-GSDMD and ASC compared with the HK-2 cells. The expression of NLRP3, NEK7, ASC, active caspase-1, and N-GSDMD was significantly enhanced (*P* < 0.05) and the inflammatory factors including IL-18, IL-1*β*, and THF-*α* were all increased in LPS-induced HK-2 cells and CLP-induced mice. Renal edema, serum Cr and BUN, and expression of KIM-1 and NGAL were significantly higher (*P* < 0.05) in CLP-induced S-AKI mice than the sham group. miR-101-3p, miR-144-3p, miR-181a-5p, miR-4262, and miR-513b-5p could inhibit NEK7. NEK7 is an interacting protein of miRNA-181a-5p. miR-181a-5p inhibits pyroptosis of the LPS-induced HK-2 cells through downregulation of NEK7. Pyroptosis of HK-2 cells promotes inflammation. miR-181a-5p inhibits pyroptosis through downregulation of NEK7 in LPS-induced HK-2 cells and CLP-induced mice. Our study indicated miR-181a-5p as a new potential therapeutic target for S-AKI therapy.

## 1. Introduction

Sepsis is a life-threatening organ dysfunction caused by the uncontrolled inflammation of the body, of which infection is the common cause [1]. Prevention of organ dysfunction is the key to the treatment of sepsis [2]. The kidney is one of the organs easily affected by sepsis [3]. The incidence of sepsis-induced acute kidney injury (S-AKI) in the intensive care unit is extremely high, and it is one of the risk factors for death in patients with sepsis [4]. S-AKI has high morbidity and mortality especially in young children and old adults, coming with heavy burdens both on the health and economy [5, 6]. As defined by KDIGO (kidney disease: improving global outcomes), S-AKI is characterized by an increase in serum creatinine (Cr) within 48 hours and extremely low urine output and manifested as a clinical syndrome of water electrolyte and acid base balance disorders and azotemia [7].

The mechanisms underlying pathophysiological change of S-AKI have not been completely illuminated, leading to nonspecific therapies [8]. Microcirculatory dysfunction, inflammatory response, and metabolic reprogramming may be associated with S-AKI [9–11]. During sepsis, pathogenic bacteria invade the body and release pathogen-associated molecular patterns (PAMPs), such as lipopolysaccharide (LPS), DNA, and lipoteichoic acid, which combine with the body's pattern recognition receptors (PRR) to initiate the body's immune response and subsequent pro- and anti-inflammatory responses [12]. In addition, after this process is initiated, certain cells in the body are bound to be damaged or destroyed, which in turn will release cellular contents such as damage-associated molecular patterns (DAMPs). DAMP can also combine with PRR, thereby expanding the body's immune response and inflammatory response.

Different from cell apoptosis, pyroptosis is essentially an innate immune response that can be triggered by the process by which host PRR recognize structures, DAMPs, and conserved PAMPs [13]. There are two pathways found to trigger pyroptosis [14]. One is the canonical inflammasome pathway, that is, the inflammasome pathway mediated by caspase-1, NLRP1, NLRP3, NLRC4, AIM2, and pyrin-inflammasome pathway [15]. The other is noncanonical inflammasome pathway, that is, the inflammasome pathway mediated by caspase-4/5/11 [16]. Pyroptosis is a novel programmed cell death, also known as GSDMD-mediated programmed necrosis, triggered by disturbance of extracellular or intracellular homeostasis associated with innate immunity [17]. Morphologically, pyroptosis is accompanied by features of necrosis and apoptosis. The early stages of pyroptosis produce chromatin condensation and DNA fragmentation, followed by the formation of necrotic-like cell membrane pores, cell swelling, and membrane rupture, leading to the release of cellular contents and proinflammatory mediators, including IL-1*β* and IL-18 [18].

MicroRNAs regulate gene expression, thus diverse cellular and physiological processes. Extensive studies have indicated a plethora of specific microRNAs in the pathogenesis of AKI [19]. However, only limited studies focused on the pyroptosis in the context of S-AKI. In this study, the miR-181a-5p was screened out. By establishing the LPS-induced HK-2 cell model and CLP-induced mouse model, miR-181a-5p was found to inhibit pyroptosis in S-AKI through NEK7. Our study provides a new potential therapeutic target for S-AKI therapy.

## 2. Materials and Methods

### 2.1. Cell Culture and Transfection

The human renal tubular epithelial cell line HK-2 was purchased from the Cell Bank of Chinese Academy of Sciences and cultured in DMEM medium (Invitrogen, CA, USA) containing 10% fetal bovine serum (Invitrogen, CA, USA) at 37°C with 5% CO_2_. HK-2 cells were incubated with medium containing 1 *μ*g/ml LPS (Sigma, MO, USA) for 24 h. Then, the cells were seeded in a culture plate (0.6 cm, 1 × 106 per ml) and transfected with different miRNAs (RiboBio, Guangzhou, China) by Lipofectamine 3000 (Invitrogen, CA, USA).

### 2.2. CCK-8 Assay

The CCK-8 assay was performed as reported [20]. Briefly, HK-2 cells were incubated in 96-well plates for 1–4 days. 10 *μ*l CCK-8 reagent (Dojindo, Kumamoto, Japan) was added. After 30 min, the absorbance value at 450 nm was measured and cell viability was calculated in comparison with control.

### 2.3. Immunofluorescence Staining

As previously reported, the cultured cells were incubated with fluorescently labeled N-GSDMD and ASC for 30 min [21]. Then, the cells were washed with PBS (pH = 7.4) three times. After adding DAPI (1 *μ*l), the fluorescence localization and intensity were observed by a fluorescence microscope (Nikon, Japan).

### 2.4. Western Blot

After extracting the total proteins from cells and tissues, the separation was performed by 10% SDS-PAGE [22]. Then, the proteins were transferred to the PVDF membrane, followed by blocking for 1 h. Primary antibodies including anti-caspase-1 (ab62698, 1 : 1000, Abcam), anti-ASC protein (13833, 1 : 1000, Cell Signaling Technology), anti-GSDMD (39754, 1 : 1000, Cell Signaling Technology), anti-NLRP3 (13158, 1 : 1000, Cell Signaling Technology), and GAPDH (ab8245, 1 : 3000, Abcam) and the corresponding secondary antibodies were used. A hypersensitive ECL (BiossciBio, Hubei, China) was used for detection.

### 2.5. ELISA and Serum Biochemical Examination

The blood was obtained and centrifuged for 10 min. The levels of inflammatory factors in serum including TNF-*α*, IL-1*β*, and IL-18 were measured by ELISA kits (Invitrogen, CA, USA). The levels of serum Cr and blood urea nitrogen (BUN) were examined by an automatic biochemical analyzer.

### 2.6. qRT-PCR

The TRIzol regent (Invitrogen, CA, USA) was used to isolate the total RNA, which was reversely transcribed into cDNA. Subsequently, real-time PCR was conducted using the following primers: NEK7: forward: 5′-GGCAAGATCGCCGTGTAATAATTCTTCAGGTGTTGCTGTTAACATT-3′, reverse: 5′-CTCGAAGCGGCCGGCCGCCCCGACTCAATTTTCAAAGCTAATAACAGA-3′; L-FABP: forward: 5′-GCAGAGCCAGGAGAACTTTG-3′, reverse: 5′-CCTTCCCTTTCTGGATGAGGT-3′; KIM-1: forward: 5′-CTCTAAGCGTGGTTGCCTTC-3′, reverse: 5′-TGTTGTCTTCAGCTCGGGAA-3′; NGAL: forward: 5′-GGCCAGTTCACTCTGGGAAA-3′, reverse: 5′-ACAGCTCCTTGGTTCTTCCA-3′; GAPDH: forward: 5′-AATGTGTCCGTCGTGGATCT -3′, reverse: 5′-AGACAACCTGGTCCTCAGTG-3′.

### 2.7. Dual-Luciferase Reporter Assay

The dual-luciferase reporter assay system (Promega, WI, USA) was used to detect the luciferase reporter gene [23]. The target fragments of the wild type and mutant type were constructed and integrated into the pGL3 vector. Then, the plasmids were transfected with Lipofectamine 3000 (Invitrogen, CA, USA). After 48 h, the luciferase activity was measured.

### 2.8. CLP-Induced S-AKI Mouse Model Establishment

Male C57BL/6 mice (~20 g) were purchased from Vital River (Beijing, China). As previously reported, the CLP-induced S-AKI mouse model was established [24]. The animal experiment was approved by the ethics committee of Beijing Friendship Hospital. The mice were randomly divided into two groups (*n* = 10) including the CLP group and sham group. The renal tissue of each mouse was collected 24 h later after operation. The water contents in the renal tissues were measured.

### 2.9. Hematoxylin-Eosin (HE) Staining

The renal tissues were coated by paraffin, sliced, dewaxed, and hydrated by xylene. The sections were stained with hematoxylin for 5 min and eosin for 30 s, followed by scoring of tubular damage into 0 (none), 1 (1–10%), 2 (11–25%), 3 (26–45%), and 4 (46–75%) [25].

### 2.10. IHC Staining

The immunohistochemistry (IHC) staining of the renal tissues was performed to examine the expression of caspase-1, N-GSDMD, NEK7, and NLPR3 [26].

### 2.11. TUNEL Staining

The terminal deoxynucleotidyl transferase-mediated dUTP nick end labeling (TUNEL) staining was performed to examine the cell apoptosis [27].

### 2.12. RNA Pulldown

The RNA overexpression plasmids were constructed and verified [28]. Then, the primers containing the T7 promoter were designed and amplified to obtain the transcription template. The PCR product was used as template to obtain the target RNA. The interacting proteins were enriched by magnetic bead-RNA probe complexes, followed by SDS-PAGE electrophoresis silver staining to detect the enriched proteins.

### 2.13. Statistical Analysis

Multiple databases including TargetScan database, miRDB database, miCode database, and DIANA-TarBase database were used to screen miRNAs regulating NEK7 gene expression. GraphPad Prism 8.0 (IBM, USA) was used for data analysis. Data are presented as mean ± standard deviation. The *t*-test and analysis of variance (ANOVA) were conducted to analyze the differences. *P* < 0.05 was statistically significant.

## 3. Results

### 3.1. Pyroptosis of the LPS-Induced HK-2 Cells

**Figure 1:**
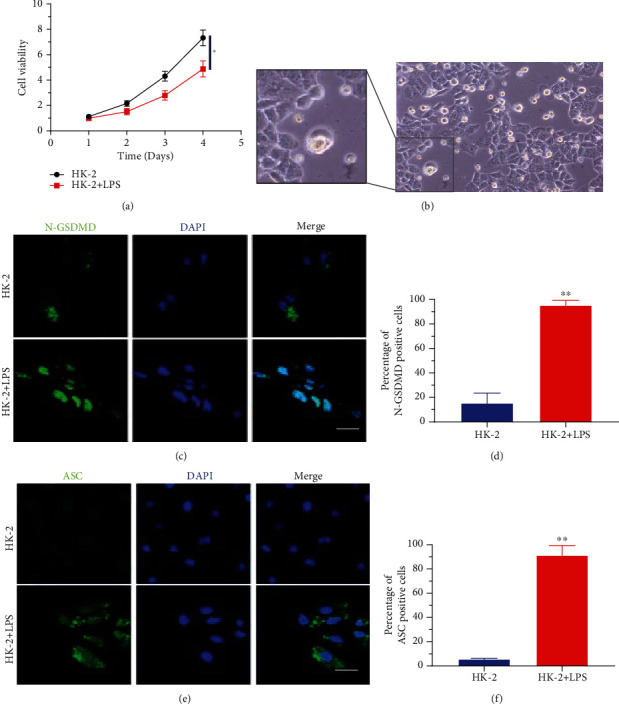
Pyroptosis of the LPS-induced HK-2 cells. (a) Cell viability of HK-2 and LPS-induced HK-2 cells. (b) Morphology of LPS-induced HK-2 cells under optical microscope (200x). (c) Immunofluorescence staining of N-GSDMD and (d) percentage of N-GSDMD-positive cells. (e) Immunofluorescence staining of ASC and (f) percentage of ASC-positive cells. ^∗∗^*P* < 0.01.

In the LPS-induced HK-2 cells, the cell viability was reduced (Figure 1(a)) and apical vacuolization was observed, indicating pyroptosis (Figure 1(b)). The fluorescent staining of N-GSDMD and ASC was performed to locate pyroptotic cells and evaluate the degree of pyroptosis. As shown in Figures 1(c) and 1(d), the LPS-induced HK-2 cells showed significantly increased (*P* < 0.01) fluorescence intensity of N-GSDMD compared with the HK-2 cells. Besides, the fluorescence intensity of ASC was significantly higher (*P* < 0.01) in LPS-induced HK-2 cells than HK-2 cells (Figures 1(e) and 1(f)). The results suggested that obvious pyroptosis was induced by LPS in HK-2 cells.

### 3.2. Pyroptosis of HK-2 Cells Promotes Inflammation

**Figure 2:**
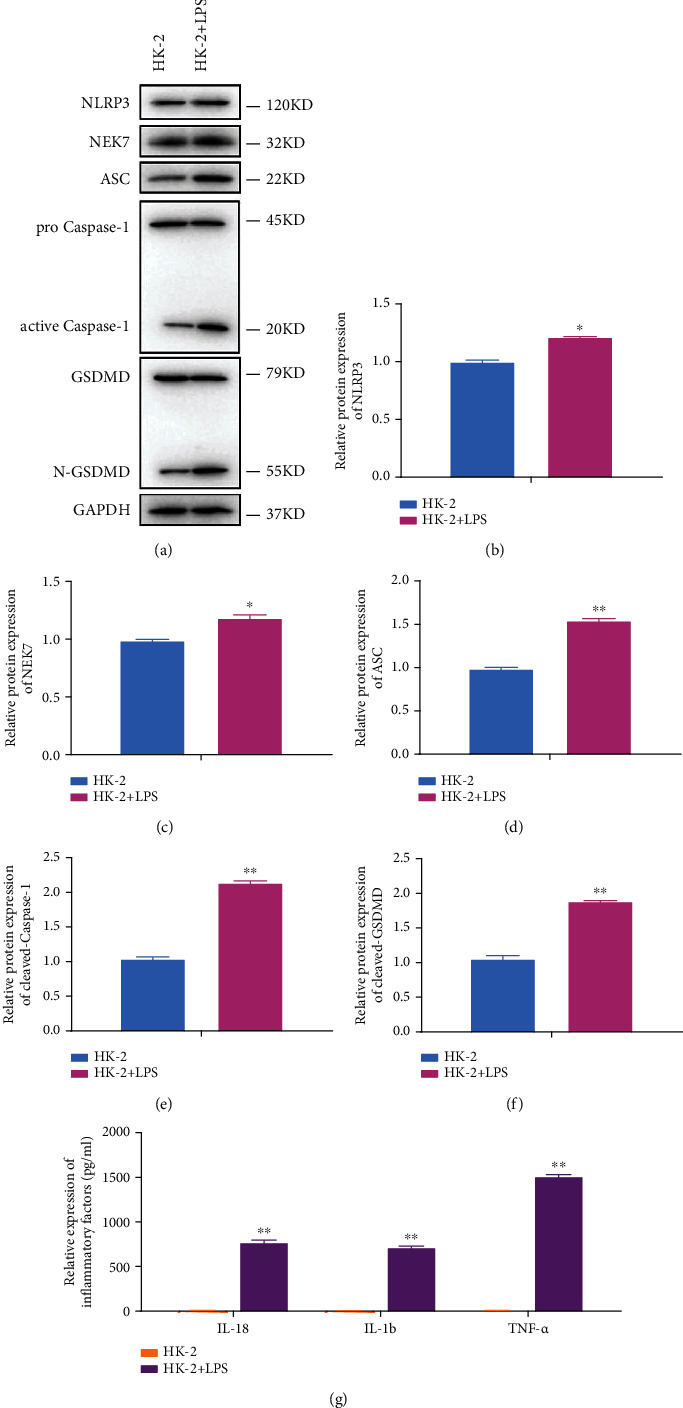
Pyroptosis of HK-2 cells promotes inflammation. (a) The protein expression of NLRP3, NEK7, ASC, active caspase-1, and N-GSDMD in HK-2 and LPS-induced HK-2 cells by Western blot, and (b–f) quantification by ImageJ. (g) The expression of inflammatory factors including IL-18, IL-1*β*, and THF-*α* by ELISA. ^∗^*P* < 0.05, ^∗∗^*P* < 0.01.

To study the effect of pyroptosis on inflammation, the expression of several important markers and inflammatory factors was examined. As shown in Figures 2(a)–2(f), the expression of NLRP3, NEK7, ASC, active caspase-1, and N-GSDMD was significantly enhanced (*P* < 0.05) in LPS-induced HK-2 cells by Western blot. Moreover, the inflammatory factors including IL-18, IL-1*β*, and THF-*α* were all increased in LPS-induced HK-2 cells by ELISA (Figure 2(g)). As is known, canonical inflammasomes are associated with NLRP3, ASC, and caspase-1, while noncanonical inflammasomes is associated with GSDMD. The results indicated that pyroptosis of HK-2 cells could activate both canonical and noncanonical inflammasomes.

### 3.3. Renal Injury in the CLP-Induced S-AKI Mouse Model

**Figure 3:**
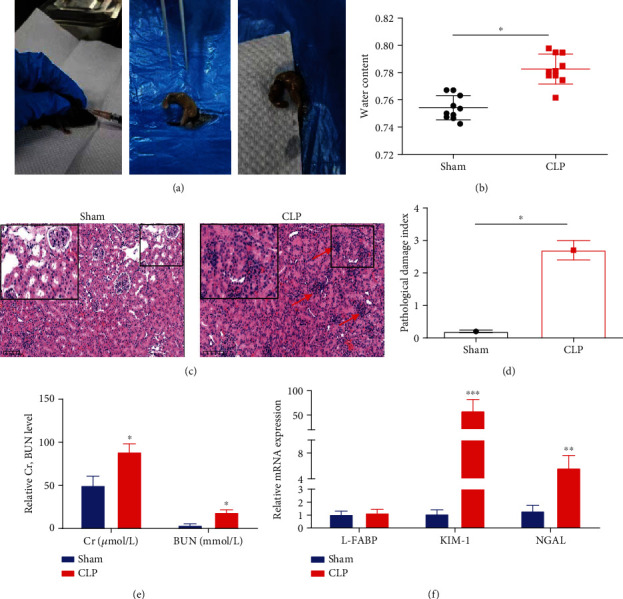
Renal injury in the CLP-induced S-AKI mouse model. (a) The CLP-induced S-AKI mouse model was established. (b) The renal water content in sham and CLP-induced mice. (c) H&E staining of renal tissues. The arrow indicates increased inflammatory cell infiltration. Scale bar = 100 *μ*m. (d) Pathological score. (e) The level of renal injury-related indicators including the serum Cr and BUN. (f) The mRNA expression of L-FABP, KIM-1, and NGAL by qRT-PCR. ^∗^*P* < 0.05, ^∗∗^*P* < 0.01, and ^∗∗∗^*P* < 0.001.

As shown in Figure 3(a) and S1, the CLP-induced S-AKI mouse model was established. The water content in CLP-induced mice was significantly higher (*P* < 0.05) than that in the sham group (Figure 3(b)), indicating increased renal edema in a CLP-induced S-AKI mouse. To further assess the extent of renal injury, the H&E staining was conducted. As shown in Figures 3(c) and 3(d), increased inflammatory cell infiltration in the renal tissues and a higher pathological damage index (2.72 ± 0.31 vs. 0.20 ± 0.04) were observed in CLP-induced mice. Furthermore, the renal injury-related indicators were measured. As shown in Figure 3(e), the Cr and BUN were significantly higher (*P* < 0.05) in CLP-induced mice than sham group. As shown in Figure 3(f), the mRNA expression of KIM-1 and NGAL was significantly increased (*P* < 0.01) in CLP-induced mice, while L-FABP remained unchanged.

### 3.4. Pyroptosis of Renal Tubular Epithelial Cells in the CLP-Induced S-AKI Mouse Model

**Figure 4:**
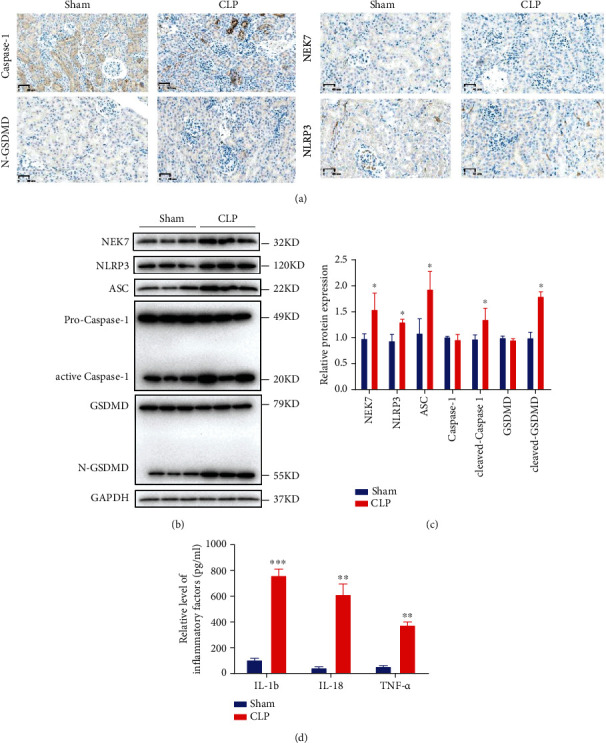
Pyroptosis of renal tubular epithelial cells in the CLP-induced S-AKI mouse model. (a) The expression of caspase-1, N-GSDMD, NEK7, and NLRP3 in sham and CLP-induced mice by IHC. (b) The protein expression of NEK7, NLRP3, ASC, active caspase-1, and N-GSDMD by Western blot, and (c) quantification by ImageJ. (d) The expression of inflammatory factors including IL-18, IL-1*β*, and THF-*α*. ^∗^*P* < 0.05, ^∗∗^*P* < 0.01, and ^∗∗∗^*P* < 0.001.

As shown in Figure 4(a), the expression of caspase-1, N-GSDMD, NEK7, and NLRP3 was significantly higher (*P* < 0.05) in CLP-induced mice than the sham group by IHC. Moreover, the protein expression was examined by Western blot as well. As shown in Figures 4(b) and 4(c), the expression of NEK7, NLRP3, ASC, active caspase-1, and N-GSDMD was significantly increased (*P* < 0.05) in CLP-induced mice. Furthermore, the inflammatory factors including IL-1*β*, IL-18, and THF-*α* were all increased in CLP-induced mice by ELISA (Figure 4(d)). These results indicated the pyroptosis of renal tubular epithelial cells in the CLP-induced S-AKI mouse model.

### 3.5. miR-181a-5p Inhibits NEK7 in HK-2 Cells

**Figure 5:**
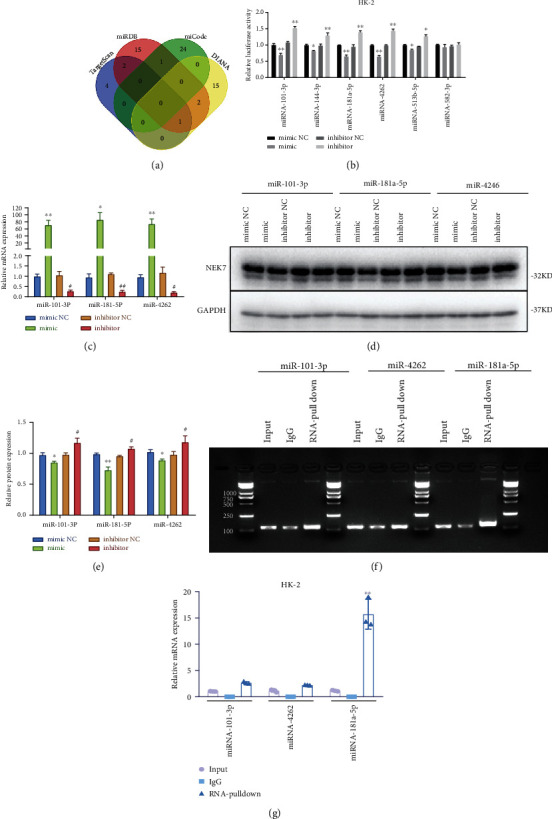
miR-181a-5p inhibits NEK7 in HK-2 cells. (a) Multiple biological databases including TargetScan database, miRDB database, miCode database, and DIANA-TarBase database to screen the miRNAs that regulate NEK7 gene expression. (b) The luciferase reporter gene detection of eight screened miRNAs. (c) The mRNA expression of NEK7 in the three top significant miRNAs by qRT-PCR. (d) The protein expression of NEK7 in the three top significant miRNAs by Western blot, and (e) quantification by ImageJ. ^∗^*P* < 0.05, ^∗∗^*P* < 0.01 compared with mimic NC; ^#^*P* < 0.05, ^##^*P* < 0.01 compared with inhibitor NC. (f) RNA pulldown of three top significant miRNAs by SDS-PAGE, and (g) quantification by ImageJ. ^∗∗^*P* < 0.01 compared with input or IgG.

The miRNAs that regulate NEK7 gene expression were screened through multiple biological information databases. According to the prediction results of TargetScan database, miRDB database, miCode database, and DIANA-TarBase database, the miRNAs supported by more than two databases that have the ability to regulate NEK7 were miR-101-3p, miR-144-3p, miR-181a-5p, miR-4262, miR-582-3p, and miR-513b-5p (Figure 5(a)). Based on the prediction of the abovementioned database, the luciferase reporter gene detection was conducted. As shown in Figure 5(b), five miRNAs including miR-101-3p, miR-144-3p, miR-181a-5p, miR-4262, and miR-513b-5p could inhibit NEK7. The expression of NEK7 in the mRNA level (Figure 5(c)) and protein level (Figure S2, Figures 5(d) and 5(e)) further confirmed this. Of note, the miR-181a-5p exhibited the strongest inhibitory effect. In order to further verify the interaction between miRNA-101-3p, miRNA-181a-5p, miRNA-4262, and NEK7, RNA pulldown experiment was performed after miRNA was transcribed and purified in vitro and the binding protein of miRNA was detected. The results showed that miRNA-181a-5p could interact with NEK7 and it is verified that NEK7 is an interacting protein of miRNA-181a-5p (Figures 5(f) and 5(g)).

### 3.6. miR-181a-5p Inhibits Pyroptosis of the LPS-Induced HK-2 Cells through Downregulation of NEK7

**Figure 6:**
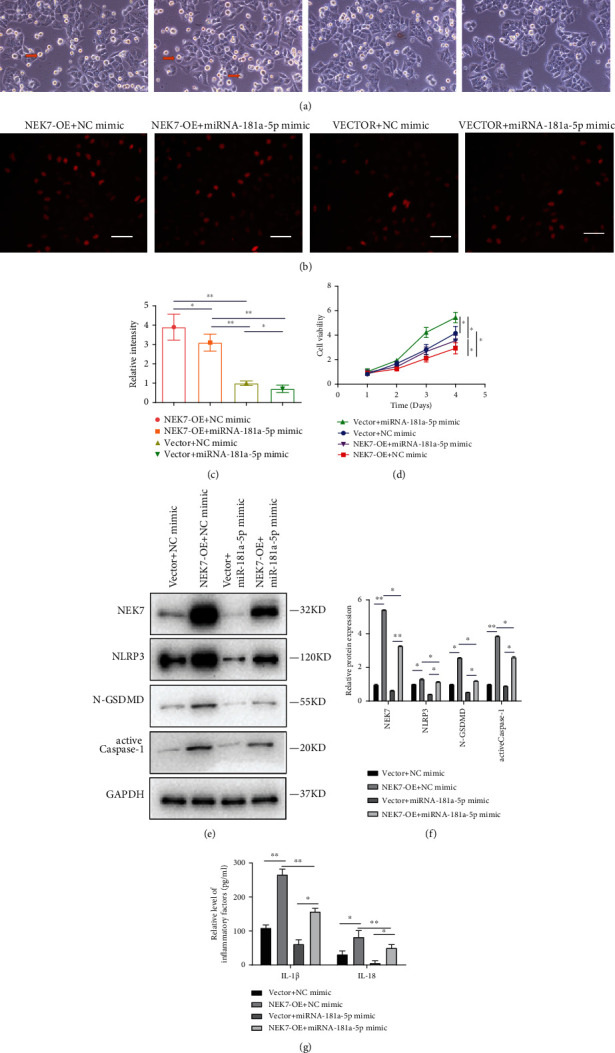
miR-181a-5p inhibits pyroptosis of the LPS-induced HK-2 cells through downregulation of NEK7. (a) Morphology of LPS-induced HK-2 cells transfected with four vectors under an optical microscope (200x). The red arrow indicates pyroptosis of the cell. (b) Cell apoptosis of LPS-induced HK-2 cells transfected with four vectors by TUNEL, and (c) relative intensity of fluorescence. Scale bar = 100 *μ*m. (d) Cell viability of LPS-induced HK-2 cells transfected with four vectors by CCK-8 assay. (e) The protein expression of NEK7, NLRP3, N-GSDMD, and active caspase-1 by Western blot, and (f) quantification by ImageJ. (g) The expression of inflammatory factors including IL-1*β* and IL-18. ^∗^*P* < 0.05, ^∗∗^*P* < 0.01.

As shown in Figure 6(a), overexpressed NEK7 promoted and miRNA-181a-5p inhibited pyroptosis of the LPS-induced HK-2 cells. Importantly, the miRNA-181a-5p could partially reverse the increased pyroptosis. Moreover, the results of cell apoptosis by TUNEL were similar (Figures 6(b) and 6(c)), indicating that miRNA-181a-5p could inhibit cell apoptosis through NEK7 as well. Consistently, higher cell viability in NEK7-OE + miRNA-181a-5p mimic than NEK7-OE + NC mimic was observed in Figure 6(d). The miRNA-181a-5p could inhibit expression of NEK7, NLRP3, N-GSDMD, and active caspase-1 and partially reverse the increased expression of these proteins induced by NEK7 (Figures 6(e) and 6(f)). The results in the mRNA level were consistent (Figure S3). The level of inflammatory factors including IL-1*β* and IL-18 also confirmed this result (Figure 6(g)).

## 4. Discussion

Accumulated evidences have suggested the microRNAs as critical regulators of renal pathophysiology and play important roles in kidney diseases such as S-AKI [29]. Juan et al. reported the exosome-mediated pyroptosis of miR-93-TXNIP-NLRP3 in S-AKI [30]. Deng et al. found that lncRNA PVT1 could modulate NLRP3-mediated pyroptosis targeting miR-20a-5p in S-AKI [31]. MicroRNA-92a-3p was identified as an essential regulator of pyroptosis in renal ischemia-reperfusion injury [32]. The miR-223-3p/NLRP3 pathway is involved in the LPS-induced AKI and inhibits HK-2 cell pyroptosis [33]. The release of microRNA-135b-5p could restrain LPS-induced pyroptotic cell death and inflammation in HK-2 cells [34]. Our study added that miR-181a-5p could inhibit pyroptosis in LPS-induced HK-2 cells and CLP-induced S-AKI mouse model through downregulation of NEK7. Yan and Huang found that miR-181a-5p overexpression could reversed the inhibitory effect of total glucosides of paeony on the pyroptosis of hypoxia/reoxygenation cardiomyocytes, indicating the relation between miR-181a-5p and pyroptosis by cell experiments [35]. Wang et al. reported that increased miR-181a-5p could aggravate kidney injury in sepsis via the PPAR*α* pathway by cell experiments [23]. This study is the first time to report the association of miR-181a-5p and pyroptosis in S-AKI by establishing the in vitro LPS-induced HK-2 cell model and in vivo CLP-induced S-AKI mouse model.

The association of S-AKI and pyroptosis remains to be better understood. There are only two studies reporting this. Zhang et al. found that downregulated IRF2 could alleviate S-AKI in vitro and in vivo [36]. Liu and Wang found that the DRP1 inhibitor could alleviate LPS-reduced S-AKI by inhibiting NLRP3 inflammasome [37]. Pyroptosis has long been considered to be caspase-1-mediated death of monocytes following infection by certain bacteria [38]. Caspase-1 is activated by different inflammasomes during various infections and immune responses. The inflammasome is a multiprotein complex with an inherent ability to induce an innate immune response by sensing damage signals and microbial attack and is present in the cytoplasm of many types of cells, including immune cells, neural cells, microglia, astrocytes, and lung endothelial cells [39]. The basic structure of most inflammasomes is composed of the nucleotide-binding oligomerization domain-like receptor (NLR) family and AIM2-like receptor (ALR) protein family as receptor proteins, apoptosis-associated speck-like protein (ASC) as adaptor protein, and caspases as effector protein [40]. Recent research has reported that GSDMD could form pores on the cell membrane, thereby increasing cell permeability and forming osmotic pressure difference inside and outside cells, which leads to cell swelling and outflow of intracellular substances, finally triggering pyroptosis [41, 42]. Our results also showed that miRNA-181a-5p could inhibit expression of NLRP3, N-GSDMD, and active caspase-1 and partially reverse the increased expression of these proteins induced by NEK7.

The NLRP3 inflammasome is important in immunity and human diseases, which can be activated by diverse stimuli [43]. NEK7 has been reported to be interacted with NLRP3 to modulate the pyroptosis in inflammatory bowel disease via NF-*κ*B signaling [44]. NLRP3-NEK7-complex formation is involved in the initiation of inflammasome assembly and pyroptosis in human macrophages [45]. The inflammasome is activated through two pathways. The first pathway is the activation of the inflammasome when the body is exposed to various endogenous and exogenous stimuli. The recruited caspase-1 is activated in the cell, cleaves IL-1*β* precursor and IL-18 precursor in the inner side of the plasma membrane, produces mature molecules of IL-1*β* and IL-18, and releases them to the outside of the cell, promoting inflammatory response and directly and rapidly activating innate immunity [46]. The second pathway is that when the body is stimulated by stimuli such as LPS, the NF-*κ*B pathway is activated, followed by nuclear translocation of NF-*κ*B to mediate activation of IL-1*β* and IL-18 precursors to produce IL-1*β* and IL-18 inflammatory factors for mediating inflammatory and immune responses [47, 48]. Since only the in vitro studies were performed, bioinformatic analysis using open-access database with sepsis population would expand the potential value of its clinical translation. Besides, gene-knockout mice can further validate the current finding. In the future study, we will continue to evaluate the outcomes of inhibiting the target molecule and clarify whether this can ameliorate the severity of AKI.

## 5. Conclusions

In conclusion, this study revealed the role of miR-181a-5p in pyroptosis of S-AKI. Pyroptosis of HK-2 cells promotes inflammation. miR-181a-5p inhibits pyroptosis through downregulation of NEK7 in LPS-induced HK-2 cells and CLP-induced mice. Our study indicated miR-181a-5p as a new potential therapeutic target for S-AKI therapy.

## Data Availability

The original data was available in a necessary request.

## Supplementary Materials

Supplementary Materials See Figure S1 for the procedure of CLP-induced S-AKI mouse model establishment. See Figure S2 for the expression of NEK7 in the protein level. See Figure S3 for the mRNA expression of NEK7, NLRP3, N-GSDMD, and active caspase-1.
