# Supplementary material for: miR-181a-5p Inhibits Pyroptosis in Sepsis-Induced Acute Kidney Injury through Downregulation of NEK7
Source: J Immunol Res. 2022 Aug 10;2022:1825490. doi: 10.1155/2022/1825490 (PMC9385359; doi:10.1155/2022/1825490)
Supplement: Supplementary Materials — See Figure S1 for the procedure of CLP-induced S-AKI mouse model establishment. See Figure S2 for the expression of NEK7 in the protein level. See Figure S3 for the mRNA expression of NEK7, NLRP3, N-GSDMD, and active caspase-1. [file 1825490.f1.docx]

Supporting information


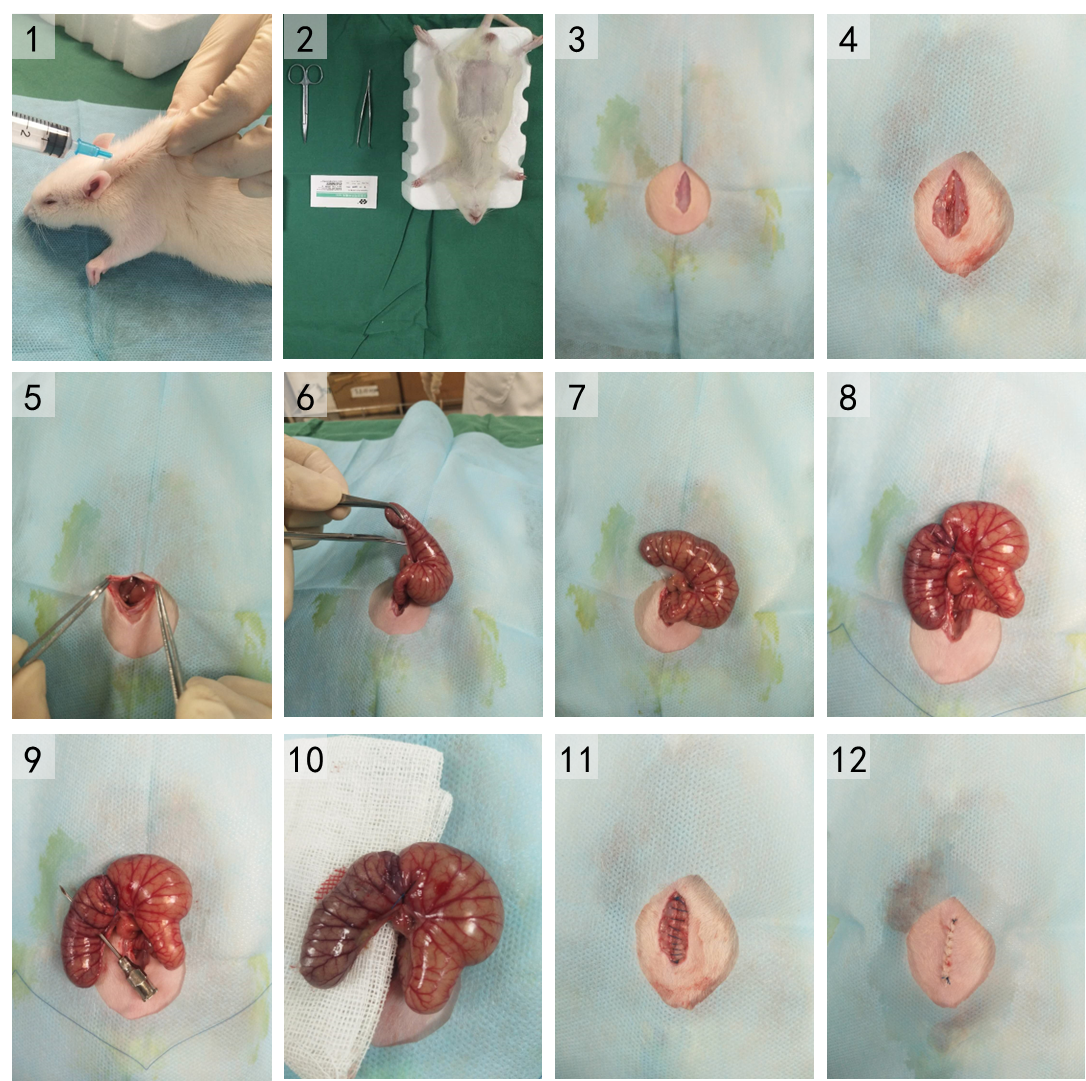


Figure S1 The procedure of CLP-induced S-AKI mouse model establishment.


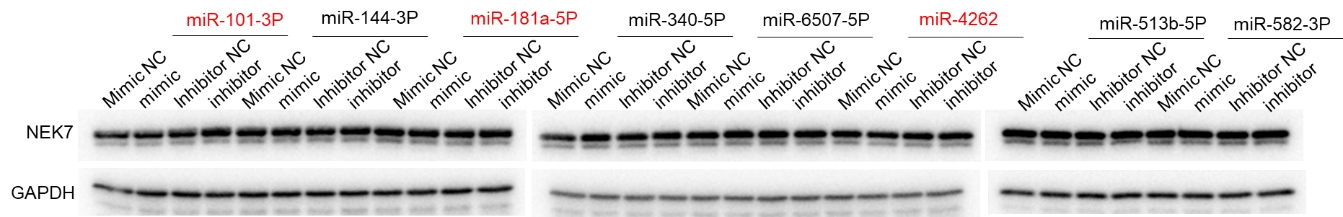

Figure S2 The expression of NEK7 in protein level.

Figure S3 The mRNA expression of NEK7, NLRP3, N-GSDMD, and active Caspase-1.
